# The application of PEF technology in food processing and human nutrition

**DOI:** 10.1007/s13197-020-04512-4

**Published:** 2020-05-08

**Authors:** Karolina Nowosad, Monika Sujka, Urszula Pankiewicz, Radosław Kowalski

**Affiliations:** Department of Analysis and Evaluation of Food Quality, University of Life Sciences in Lublin, Skromna 8, 20-704 Lublin, Poland

**Keywords:** Pulsed electric field, Food processing, Functional food

## Abstract

During the last decades, many novel techniques of food processing have been developed in response to growing demand for safe and high quality food products. Nowadays, consumers have high expectations regarding the sensory quality, functionality and nutritional value of products. They also attach great importance to the use of environmentally-friendly technologies of food production. The aim of this review is to summarize the applications of PEF in food technology and, potentially, in production of functional food. The examples of process parameters and obtained effects for each application have been presented.

## Introduction

Development of innovative food processing methods can increase the competitiveness of the food industry by improving product quality, introducing new products to the market and reducing production costs (Tiwari et al. 2009). Incorporation of pulsed electric field (PEF) technology into food production was supported by the growing consumer interest in food of high nutritional value, the demand for fresh-like products as well as food produced with the use of environmentally friendly methods (Evans and Cox 2006; Soliva-Fortuny et al. 2009). The studies showed that despite the fact that consumers have rather conservative approach and it is not always easy for them to see the benefits of novel processing technologies, they appreciate the naturalness, improved taste and high nutritional value of the products subjected to PEF (Nielsen et al. 2009; Sonne et al. 2012). As suggested by the results of studies conducted in different countries, providing detailed and reliable information about new technologies may be of key importance for increasing consumer acceptance of products obtained using novel food processing technologies (Lee et al. 2015; Galati et al. 2019; Maherani et al. 2016).

Despite many scientific studies on the principles and applications of PEF technology published so far and the fact that PEF was introduced into the food industry many years ago, this technology is still considered emerging. In the European Union there is no special legislation on food processed with PEF. In general the use of this technique is regulated by the Novel Food Regulation (EU) 2015/2283, but implementation of PEF into production does not automatically mean the food becomes “novel”. According to Article 4 of Regulation (EC) No. 258/97, a food product can be considered as novel if the production process applied causes significant changes in its composition or structure influencing nutritional value, metabolism or level of undesirable substances. The studies showed that, for instance, in case of liquid products such as oils, juices and beverages containing juices no significant decreases in content of health-beneficial compounds have been observed as a result of PEF treatment (Guderjan et al. 2005; Zulueta et al. 2007; Salvia-Trujillo et al. 2011; Morales-De La Peña et al. 2012; Vallverdú-Queralt et al. 2012). The party who wants to market the food is responsible for clarifying its regulatory status with the national food authority body. Decision on food novelty is based on the procedures described in the Commission Implementing Regulation (EU) 2018/456 and safety assessment must be carried out as a part of the authorisation process. The use of novel processing technologies has the potential to reduce the environmental impact of food production and increase food safety, so their use is regulatory encouraged in the EU (Regulation (EU) 2015/2283). Regulations concerning novel foods exist also in Canada, New Zealand/Australia, China, and Brazil but the definition of “a novel food” may differ (Magnuson et al. 2013). In the United States, prior to 2002, the Food and Drug Administration considered pasteurization as a thermal treatment but in September 2004, the USDA National Advisory Committee on Microbiological Criteria for Foods (NACMCF) redefined the term “pasteurization” as “any process, treatment, or combination thereof, that is applied to food to reduce the most microorganism(s) of public health significance to a level that is not likely to present a public health risk under normal conditions of distribution and storage” allowing methods such as PEF to be used (NACMCF 2004). PEF has been used for the commercial pasteurization of juices in compliance with the mandates of FDA’s juice HACCP regulations (21 C.F.R. 120). The juice processors also have to implement sanitation and Good Manufacturing Practices during production of juice and juice products. According to FDA the processes of production should meet a performance standard of 5 log reduction of the most resistant pathogen (FDA 2000).

Pasteurization of liquid foods still remains the main purpose of using PEF technology. The lethal effect of PEF on various vegetative bacteria, mold, and yeast can be strengthen by combining with other physical methods, such as UV radiation (Gachovska et al. 2008), high intensity light pulses (HILP) (Caminiti et al. 2011), ultrasound (Aadil et al. 2018), high pressure carbon dioxide (Pataro et al. 2010) and manothermosonication (Palgan et al. 2012).

## General overview: advantages and disadvantages of PEF

PEF is a method that uses electric waves with high voltage amplitude. Short electrical impulses (from microseconds to milliseconds each) of high voltage (typically 10–80 kV/cm) are supplied to the product placed between the electrodes in the chamber (Deeth et al. 2008). Depending on the properties of the processed food product and the effects to be obtained, the process conditions such as electric field strength (kV/cm), pulse frequency, pulse width, shape of the pulse wave and exposure time (related to the flow rate and volume of fluid in the electrode chamber) can be modified suitably. For instance, the range of electric field strength 0.1–1 kV/cm causes reversible permeabilization of plant cells, 0.5–3 kV/cm—irreversible permeabilization of plant and animal tissue, 15–40 kV/cm—irreversible permeabilization of microbial cells (Tsong 1996).

In the last decade, one of the main fields of research in the scope of alternative energy-saving processes has been the application of PEF as a non-thermal method in food processing (Soliva-Fortuny et al. 2009). Nevertheless, it should be mentioned that the energy of the electric pulses generates heat due to Joule heating so cooling is necessary to maintain a low temperature of the processed product during PEF treatment. On the other hand, this phenomenon can be applied for a gentle preservation process. The combination of high temperature and PEF membrane electroporation improves also the inactivation efficiency (Jaeger et al. 2010b).

Most of the research on the use of PEF relates to inactivation of enzymes and microorganisms. High voltage impulses break the cell membrane making it permeable to small molecules, which causes the cells to begin to swell and break (Zimmerman 1986). PEF can be used for liquid and semi-solid products e.g. soups, liquid eggs or fruit juices (Qin et al. 1995). Fruit juices processed with this technology were introduced to the US market in 2005 (Ravishankar et al. 2008). In the case of solid products, PEF technology has found application mainly in potato processing industry. Potatoes can be subjected to PEF immediately after peeling and before the cutting step (Faridnia et al. 2015a) or in a form of slices. The effect of PEF is a change in the structural integrity of tissues, which results in more controlled release of intracellular compounds such as reducing sugars or amino acids involved in Maillard reactions, and therefore reduces acrylamide content in cooked or fried potato products (Jaeger et al. 2010a, b; Janositz et al. 2011; Genovese et al. 2019). Potatoes treated with PEF also have a more uniform color and absorb less oil during frying (Ignat et al. 2015; Liu et al. 2018a, b). Another effect of PEF is a softer texture that facilitates potato processing, e.g. cutting (Lebovka et al. 2004) and a significant decrease in drying time of potato discs (Fauster et al. 2018).

Although this technology has been investigated extensively and there are several dozen commercial PEF systems working around the world, the majority of the obtained results refer to the experiments carried out at laboratory scale. The pulsed electric field technology itself is generally considered to be safe for humans, because no dangerous chemical reactions have been detected (Frewer et al. 2011). However, the results of some studies indicate that electrode material constituents (e.g. Fe, Cr, Ni, Mn) are released to the liquid food samples due to corrosion (Roodenburg et al. 2005; Pataro et al. 2014). This problem may be overcome by application of carbon electrodes (Toepfl et al. 2004). According to Pataro et al. (2014), some electrical parameters such as pulse frequency and the composition of the processed product (e.g. presence of halides) affect the amount of metal released from the electrodes. Undoubtedly, further research is needed to determine the optimal conditions for PEF treatment on an industrial scale, as well as electrode material and geometry, so that undesirable electrode reactions will be eliminated or at least minimized.

**Fig. 1:**
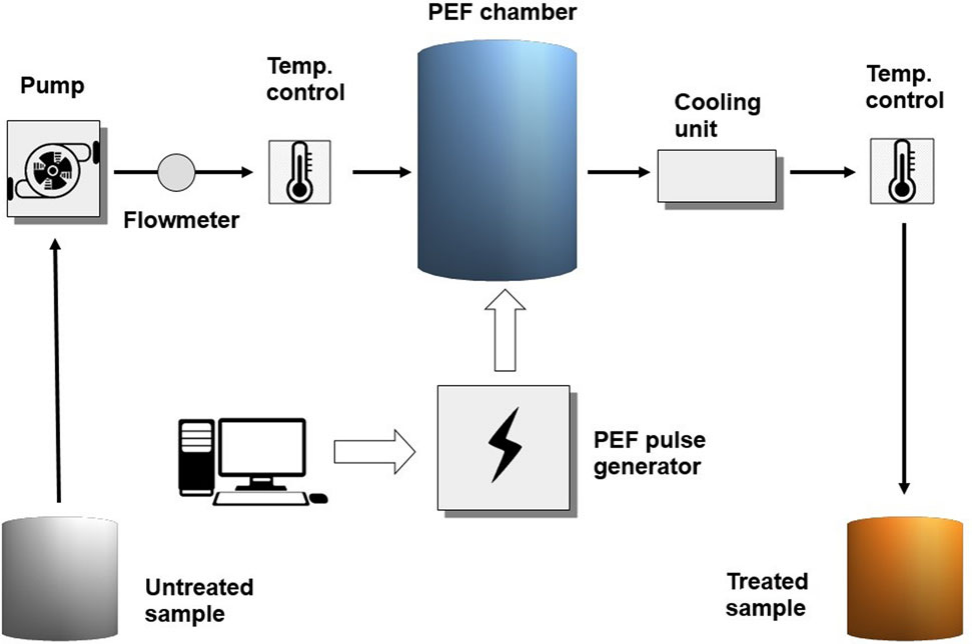
A typical PEF unit used in food processing

A typical PEF unit is composed of a few basic components: high-voltage pulse generator, treatment chamber, fluid-handling system, control and monitoring devices (Fig. 1). The first component supplies the high voltage pulses with required shape, duration and intensity. The generated pulses are applied to a pair of electrodes present in the treatment chamber and the treated product is placed between them. Depending on the type of the treated product (solid, semisolid, liquid, semiliquid), the treatment chambers can be divided into batch treatment chambers and continuous treatment chambers. The latter type is very convenient for industrial processes because allows liquid and semi-liquid products to be pumped through the chamber. The process is controlled by a central computer which is used for setting the parameters, controlling the operation of pump and gathering data from the probes placed inside the chamber (Barbosa-Canovas et al. 2004). In liquid products processed with PEF the serious problem is non-uniformity of electric field distribution inside the treatment chamber caused by its configuration, presence of bubbles/impurities and thermophysical properties of the product itself (Zhang et al. 1995). As a result some parts of the liquid volume can be undertreated (often in central or dead spaces) or overtreated (often in boundary regions). Achieving of electric field uniformity is particularly important in the case of cold pasteurization, because during this process all microorganisms present in the liquid should be exposed to the same electric field strength and the same number of pulses. To overcome this problem, treatment chambers with parallel plate electrode configurations or multiple PEF treatment chambers placed in series can be used (Buckow et al. 2013).

## Mechanism of pulsed electric field

The application of PEF on plant, animal or microbial cells disturbs transiently or permanently the integrity of cell membrane increasing its permeability, however the mechanism of PEF activity has not been fully understood. Until now, several theoretical models have been suggested, but there is still no evidence of PEF’s action regimen at the cellular level (Soliva-Fortuny et al. 2009). Experimental evidence suggests that aqueous hydrophilic pores are formed as a consequence of rearrangements of the membrane components such as water and lipids, induced by long and intense electrical pulses (Weaver 2003). It is not possible to observe directly pores of sizes in nanometers with conventional methods e.g. electron microscopy. However, nowadays computational methods (molecular dynamics simulations) can be used to model the effect of electric field in cell membrane (Leach 2001) The simulations conducted by Tieleman (2004) have evidenced that the electroporation process takes place in two stages: (1) water molecules organized in single wire penetrate the hydrophobic core of the bilayer; (2) the water wires grow in length and expand into water-filled pores, which are later stabilized by reorganization of lipid molecules.

When a biological cell is considered as an electrolyte surrounded by an electrically insulted shell (the cytoplasm surrounded by the plasma membrane) and it is exposed to an external electric field, this results in occurrence of induced transmembrane voltage (Kotnik et al. 2010). Under physiological conditions there is a ionic gradient across the membrane resulting from the work of sodium–potassium pumps and potassium leak channels. Its value depends on cell type and typically ranges from − 80 to − 40 mV (Kotnik and Miklavčič 2006). Permeabilization of cell membrane is a local process and takes place when the transmembrane potential difference induced by electric field reaches 250 mV. This part of cell surface becomes highly permeable for small charged molecules such as DNA or metal ions (Teissié and Tsong 1981). Diffusion is observed mainly after the pulse and lasts for seconds and minutes (Gabriel and Teissie 1997). Permeabilization is dependent on field strength, pulse parameters (amplitude, duration, pulse number and repetition rate), membrane composition, surrounding media, temperature, cell size and shape and its orientation to the electric field lines (Valič et al. 2003). This phenomenon can be detected e.g. with the use of fluorescent indicators such as propidium iodide (PI) (Sadik et al. 2013) or by means of electrical measurements (bio-impedance or micro-electrodes techniques) (Silve et al. 2011, 2017). The uptake of the indicator by the cells is the evidence of membrane permeabilization. When the operation of the electric field stops, the membrane defects become sealed, and the cells retain the introduced molecules or ions. It depends on the time of exposure and the intensity of the electric field. Resealing can last from a few second to several hours, depending on temperature. For example, at 37 °C the membrane defects close in a few seconds, at 4 °C in a few minutes and when cells are maintained on ice—several hours are needed. When the field strength exceeds the critical value significantly, the process is irreversible and can lead to cell destruction (Golzio et al. 2002).

## Applications of PEF in food processing

The PEF technique was of interest already in the twentieth century. At the beginning of the 1990s, a milk pasteurization method was developed in which a low-frequency alternating electric field was used. In 1960 a German engineer Doevenspeck patented a method that used high-voltage electric waves to break down the structure of the cells of food materials (Toepfl et al. 2006). Processing factors such as electric field strength, pulse shape, pulse width, treatment time, pulse frequency and polarity, temperature, treatment in batch or continuous flow system are critical factors determining the efficiency of PEF technology in food processing. Optimization of PEF parameters is required for each specific application of pulsed electric field. Some examples are presented in Table 1.

**Table 1:** Examples of process conditions and effects of using PEF in food processing

Material	PEF parameters	Effect of PEF	References
*Drying*			
Basil (*Ocimum basilicum* L.) leaves	65 pulses of 650 V/cm, 150 µs pulse width, 760 µs between pulses	Drying times reduced 57% for air drying, 33% for vacuum drying and 25% for freeze drying	Telfser and Gómez Galindo (2019)
Parsnip and carrot	20 μs, 50 Hz, 0.9 kV/cm, after 1000 pulses	Drying time reduced to 28% at 70 °C and to 21% at 60 °C, compared to the untreated samples	Alam et al. (2018)
Carrot	Pulse number 10, 50 and 100; 1,85 and 5 kV/cm; 5,63, 8 and 80 kJ/kg	Drying time reduced up to 8.2%. Decrease of sample lightness up to 25.3%	Wiktor et al. (2016)
Potato tissue	300–400 V/cm	Decreasing the drying temperature approximately on 20°	Lebovka et al. (2007)
*Extraction*			
Citrus fruits and peel (orange, pomelo and lemon)	3 kV/cm—fruits 10 kV/cm—peel	Increased yield of juice by 25% for oranges, 37% for pomelos and 59% for lemon, improved extraction of polyphenols to 50%	El Kantar et al. (2018)
Fruit juice with the addition of stevia	30 kV/cm for 230 μs	The retention of ascorbic acid increased by over 74%. The enhancement of anthocyanins and carotenoids extraction	Carbonell-Capella et al. (2017)
	40 kV/cm for 230 μs	The highest content of hydroxymethylfurfural	Carbonell-Capella et al. (2017)
	21 kV/cm 300 μs with 2.5% stevia	The highest content of bioactive compounds and sweetening properties obtained with minimal color changes	Carbonell-Capella et al. (2017)
Blueberry fruits (*Vaccinium myrtillus* L.)	1,3 and 5 kV/cm, 10 kJ/kg	Increasing the juice yield (+ 28%) compared to the untreated sample. The juice obtained had a significantly higher total phenolic content (+ 43%), total anthocyanin content (+ 60%) and antioxidant activity (+ 31%)	Bobinaitė et al. (2015)
*Freezing*			
Baby spinach leaves	Two trains of bipolar, rectangular pulses with amplitude of 350 V, with 10 s interval between trains. Each train consisted of 500 pulses of 200 μs pulse width and 1600 μs of space between the pulses (frequency 500 Hz)	Improved freezing tolerance by applying vacuum impregnation and PEF in the presence of cryoprotectants	Demir et al. (2018)
Apple tissue	800 V/cm, pulse duration 1000 μs, time interval 100 ms, 10 pulses	Acceleration of cooling processes; good preservation of the macro-shape, inhibition of shrinking, development of large pores in the electroporated tissue	Parniakov et al. (2016a)
Beef muscle	1,4 kV/cm, 20 µs, 50 Hz, 250 kJ/kg (combined with freezing and thawing)	Microstructural changes in meat tissue, improved tenderness and purge loss	Faridnia et al. (2015b)
*Preservation*			
Fresh berries	2 kV/cm, pulse width 1 μs and 100 pulses per second for 2, 4 and 6 min + disinfectant solution (60 ppm peracetic acid [PAA])	The reduction of *E. coli* and *Listeria innocua* without changing the color and appearance of blueberries The softening of the berry structure Conc. of anthocyanins and phenolic compounds increased by 10 and 25%, respectively	Jin et al. (2017)
Peptides isolated from pine nuts	1800 Hz, 15 kV/cm	No changes of the amino acid sequence	Lin et al. (2017)
Milk	25.7 kV/cm for 34 μs after heating to 55 °C and maintained for 24 s and heat treatment at 63 °C for 30 min or at 73 °C for 15 min	Inactivation of alkaline phosphatase. Reduced xanthine (30%) and plasmin oxidase (7%) activity	Sharma et al. (2017)

### Drying

Pre-treatment of the sample with PEF in order to destroy the cell structure reduces its resistance to diffusion and the mass and heat transfer rates between the cells and their surroundings increases (Barba et al. 2015). Research on the impact of initial PEF treatment on drying kinetics as well as changes in color and texture in sliced parsnip and carrot was carried out by Alam et al. (2018). The drying time was reduced to 28% at 70 °C and to 21% at 60 °C compared to the untreated samples. Wiktor et al. (2016) observed that drying time of the PEF-treated carrot samples was reduced up to 8.2%, the effective water diffusion coefficient increased up to 16.7%, and samples after drying exhibited higher lightness and redness in comparison to the intact tissue. Effect of a PEF pre-treatment on drying of onions was investigated by Ostermeier et al. (2018). The study revealed that a rising electric field strength up to 1.07 kV/cm caused an increase of the cell disintegration which facilitated the moisture release to the surface of the product. The higher diffusion led to a 30% reduction in drying time for PEF pre-treated onion samples dried at 45 °C. Telfser and Gómez Galindo (2019) studied the effect of reversible permeabilization as pre-treatment before air drying at 40  °C, vacuum drying and freeze drying of basil (*Ocimum basilicum* L.) leaves. The application of PEF shortened the drying time by 57% for air drying, 33% for vacuum drying and 25% for freeze drying. Samples which were PEF-treated and vacuum dried were found to be the closest to fresh leaves regarding colour and smell determined by sensory panel. Application of PEF accelerates also the drying of carrots, potatoes, apples, coconuts or paprika (Ade-Omowaye et al. 2001).

### Freezing

Freezing food has one major disadvantage—the formation of ice crystals can destroy the tissue so that after thawing the products (for example soft fruits, leafy vegetables) lose their shape and become sodden. In this form they are not accepted by the consumers. It has been demonstrated that pulsed electric fields can be used to improve freezing tolerance of baby spinach leaves. PEF was applied with vacuum impregnation in the presence of cryoprotectants such as trehalose, sucrose, glucose, and fructose. The combination of these methods caused that leaf cells remained viable and the leaves retained turgor after the freezing and thawing cycle (Demir et al. 2018). Carrot discs treated with PEF after soaking in different cryoprotectant and texturizing agents had higher firmness after thawing than control sample (Shayanfar et al. 2014). Similar studies were carried out for potato strips. The results showed that PEF treatment by itself (without texturizing and antifreeze agents) was not a suitable pre-treatment method but when it was applied with CaCl_2_ and trehalose potato strips maintained structural integrity, firmness and colour after thawing (Shayanfar et al. 2013). Interestingly, there was no improvement in texture of strawberries frozen and thawed after the application of PEF coupled with vacuum infusion and cryoprotectants. However, such treatment enhanced the color retention of thawed fruits (Velickova et al. 2018).

Application of PEF technology together with freezing or freeze-drying affects freezing time and rate. For example, the study of Jalté et al. (2009) showed that PEF pre-treatment can reduce the freezing time, increase the rate of freeze-drying and improve quality of the freeze-dried potato. Similarly, Wiktor et al. (2015), who studied influence of PEF on freezing and thawing of apple tissue, observed that the total freezing time and the total thawing time were reduced by, respectively, 3.5–17.2% and 71.5%. Similar results were reported by Ben Ammar et al. (2011) and Al-Sayed et al. (2018). The authors concluded that electroporation of multicellular tissues led to better connections between intra- and extracellular content allowing increased probability of ice nucleation and faster ice propagation after freezing and correspondingly shortening the freezing time.

### Food preservation

Food deterioration may be caused by several factors such as microorganisms development and activity of endogenous enzymes. The PEF technology, compared to the traditional pasteurization method, not only inactivates pathogenic microorganisms but also enzymes in some extent, minimizes the loss of the original taste, color, texture, nutrients and other thermolabile compounds found in food (Syed et al. 2017). For this reason, it is a promising supplement or substitute for traditional thermal pasteurization. PEF can be successfully used for liquid products with low viscosity and electrical conductivity, e.g. milk and juices.

#### Microbial inactivation

Milk and dairy products are processed using various thermal methods to make them safe for human consumption. Incorrect pasteurization of milk causes spoilage of the product and formation of pathogenic bacteria such as *Escherichia coli*, *Listeria* spp. and *Pseudomonas*. The treatment in which high temperatures are used causes nutrient losses (Ercolini et al. 2009). Pulsed electric field not only inactivates bacteria at low temperatures, but also affects minimally the nutritional and sensory properties of the food product. PEF causes inactivation of Gram-negative and Gram-positive bacteria in a whole milk already at 50 °C (Sharma et al. 2014). Milk that has been thermally preserved can be microbiologically stable for 21 days when stored at 4 °C. However, heat causes unfavorable effects such as: damage to the creaming properties, non-enzymatic browning, degradation of lactose, denaturation of whey proteins (Fox et al. 2015). PEF technology can be used synergistically with heat, antimicrobial agents, membrane filtration and ultraviolet radiation in order to increase the effectiveness of bacterial inactivation and prolonging the period of consumption. In one of the studies of Sharma et al. (2017), milk samples were subjected to PEF with the following parameters: 25.7 kV/cm for 34 μs after heating to 55 °C and maintained for 24 s and heat treatment at 63 °C for 30 min or at 73 °C for 15 min. Inactivation of alkaline phosphatase was comparable in all samples. The PEF-treated sample initially exhibited reduced xanthine (30%) and plasmin oxidase (7%) activity, however, after 21 days of refrigeration storage, these parameters were similar to the milk sample not processed at all. During storage in all milk samples, lipolytic activity increased and the pH level dropped. Hemar et al. (2011) reported that PEF has no impact on whey proteins and milk pH, but it can affect the viscosity and particle size when milk is treated with high field strengths.

Jin et al. (2017) studied how PEF affects the native microflora and the population of *E. coli Listeria innocua*, which have been artificially grafted on blueberries. The combination of PEF and PAA (60 ppm peracetic acid) resulted in the reduction of *E. coli* and *Listeria innocua*, but it did not change the color and appearance of blueberries. The only disadvantage of the process was the softening of the berry structure. Anthocyanins and phenolic compounds increased by 10 and 25%, respectively. Palgan et al. (2012) combined PEF and manothermosonication (MTS) to reduce *Listeria innocua* in a milk based smoothie. The study showed that the application of MTS followed by PEF was the most effective in inactivating *L. innocua* causing a mean reduction of 5.6 log cfu/ml.

#### Spore inactivation

PEF processing seems to have no effect on endospores although some publications describe a certain level of spore inactivation achieved if the necessary harsh conditions are applied. Spores present a higher resistance to PEF than vegetative cells due to their small sizes, low permeability, dehydration and mineralization (Setlow 1995). Therefore, at present, PEF treatment alone can be applied for pasteurization but not for sterilization purposes. However, combined application of PEF with other methods e.g. thermal treatment can lead to successful inactivation of endospores. For instance, Siemer et al. (2014) reported a 3 log cycles inactivation of *B. subtilis* spores under the following process conditions: electric field strength of 9 kV/cm, inlet temperature of 80 °C, the addition of 10% sugar to medium. Similarly, Reineke et al.(2015) achieved a 4.67 log_10_ inactivation of *B. subtilis* spores in saline water when they applied the process conditions: 70 °C with a flow rate of 5 l/h, a frequency of 150 Hz, an energy input of 226.5 kJ/kg.

#### Enzyme inactivation

Enzymes are less sensitive to the action of PEF than microbes so more intense PEF treatments are required for their inactivation (Ho et al. 1997), but the mechanism of this phenomenon is still not well understood. Probably both electrochemical and thermal effects, which are associated with PEF, cause the changes in the structure and conformation of enzymes leading to their inactivation (Terefe et al. 2013). In the case of grape juice, which is susceptible to the action of many enzymes, PEF did not significantly affect its physicochemical and sensory characteristics but it reduced the activity of polyphenyl oxidase and peroxidase. The duration of PEF action, its intensity and frequency had a significant effect on the relative activity of selected enzymes, which was abolished along with the increase of the above-mentioned parameters (Marselle´s-Fontanet and Martin-Belloso 2007).

### Extraction of bioactive compounds

Extraction is one of the most commonly used processes in the industry to obtain valuable compounds and usually it involves chemical and/or thermal treatment of a sample. Numerous studies report that application of pulsed electric field for extraction can enhance its efficiency, reduce the extraction time and minimize any damage to the extracted nutrients. PEF has been used to improve the extraction of intracellular compounds from fruits and vegetables. Luengo et al. (2013) reported that amount of polyphenols extracted from tomatoes and grapes increased after treatment with PEF. The use of this technique enhanced also extraction of polyphenols from borage (*Borago officinalis* L.) leaves and increases their antioxidant activity. In addition, it also reduced the extraction time, and the increase in pulse intensity was proportional to the amount of polyphenols extracted, as well as to their antioxidant properties (Segovia et al. 2014). Soliva-Fortuny et al. (2017) studied the effect of PEF on the content of phenols, flavonoids and flavan-3-ol as well as on the antioxidant capacity of apples stored at different temperatures (4 and 22 °C) for 48 h. The maximum increase in the total phenol content (13%) and flavone-3-ol (92%) was observed in apple treated with the mildest electric field parameters. The antioxidant activity was also higher in apples subjected to PEF (by 43%) in relation to the untreated samples. Liu et al. (2018a, b) investigated the effect of PEF on the extraction of water-soluble phenolic compounds from onion as well as the antioxidant activity of the extracts. Results indicated that the yield of water-soluble phenolic and flavonoid compounds extracted from onion significantly increased after PEF treatment following water extraction, by 2.2 and 2.7 times, respectively, in comparison to control. The authors noted that the antioxidant activity of extracts increased with the increase in electric field intensity and treatment time. El Kantar et al. (2018) investigated the effect of PEF on citrus fruits (orange, pomelo and lemon). Fruits and peel were treated with a pulsed electric field at a field voltage of 3 kV/cm and 10 kV/cm, respectively. PEF processing increased the yield of juice by 25% for oranges, 37% for pomelos and 59% for lemon, and improved the extraction of polyphenols to 50%.

PEF is an ideal method used to enhance the extraction process of various intracellular compounds, e.g. sugar from sugar beet (Lopez et al. 2009), phytosterols from maize germs (Guderjan et al. 2005). One of the benefits of using PEF is also obtaining high purity of fruit juices (Lebovka et al. 2003). However, the use of too high intensity can lead to destruction of the cell membrane, cell turgor and may have an adverse effect on the viscosity and elasticity of plant tissue (Lebovka et al. 2004). The ability of PEF to inactivate microorganisms and induce the permeabilization of eukaryotic cells without a significant increase in the temperature of the product can be used in the process of wine production to improve its quality. The low energy consumption and short processing time required to permeabilize grape skin cells are the key advantages of using PEF in obtaining wines with a high content of phenolic compounds. The high concentration of polyphenols helps in stabilizing the color and improves the quality of the wine during the aging process (Boulton 2001). Phenolic compounds also have pro-health activities (e.g. antioxidant and pro-inflammatory properties). PEF does not affect the change in the taste, color or nutritional value of grape must and wine. It also facilitates the growth of active dry wine yeast that is added to the grape must to provide a faster fermentation process. PEF also reduces the amount of SO_2_, which spoils the quality of wine (Puertolas et al. 2010).

The fruit juice with the addition of stevia was processed by means of PEF to study the effect of this technology on bioactive compounds and steviol glycosides. PEF treatment resulted in the retention of ascorbic acid by over 74%, the enhancement of anthocyanins and carotenoids extraction. The best results were obtained at 30 kV/cm for 230 μs. At the highest voltage of 40 kV, the highest hydroxymethylfurfural content was found. With PEF carried out at 21 kV/cm during 300 μs with 2.5% stevia, the highest content of bioactive compounds and sweetening properties was obtained with minimal color changes (Carbonell-Capella et al. 2017).

Lin et al. (2017) investigated the mechanism of improving the antioxidant properties of peptides isolated from pine nuts using PEF. Radical inhibition of DPPH and cellular antioxidant activity (CAA) were used to assess the antioxidant activity of peptides. The structure of electroporated peptides was analyzed by medium-infrared spectrophotometry (MIR) and circular dichroism (CD). The capture of DPPH radicals increased significantly (89.10% ± 0.20% to 93.22% ± 0.09%) under PEF treatment conditions. The pulse frequency was 1800 Hz and the electric field voltage was 15 kV/cm. PEF did not change the amino acid sequence of Gln-Cys-His-Lys-Pro, Gln-Cys-His-Gln-Pro, Lys-Cys-His-Gln-Pro.

### Starch modification

Pulsed electric field can be used for modification of potato, corn, wheat, waxy rice, and cassava starches (Han et al. 2009; Hong et al. 2018; Li et al. 2019; Zeng et al. 2016). The researchers observed re-arrangement and destruction of starch molecules as well as a decrease in gelatinization properties, viscosity and crystallinity along with the increase in field strength (1.25–5 kV/cm and 30–50 kV/cm). Application of PEF affected starch digestibility increasing level of rapidly digestible starch in potato, wheat and pea starches (PEF intensity of 2.86, 4.29, 5.71, 7.14, and 8.57 kV/cm, 600 Hz of pulse frequency, 6 μs of pulse width) (Li et al. 2019) and in waxy rice starch (30, 40 and 50 kV/cm) (Zeng et al. 2016). Hong et al. (2018) reported that such starch modification methods like acetylation can be significantly enhanced by PEF treatment (PEF parameters: pulse frequency of 1000 Hz; field intensity of 1.25, 2.50, 3.75, and 5.00 kV/cm; pulse duration time of 40 μs). The use of PEF to support the starch modification methods can enhance the process’s efficiency, reduce reaction time and save reagents.

### Waste valorisation in the food industry

Food industry generates huge quantities of by-products and wastes, which are problematic because their disposal is associated with environmental and health related issues. On the other hand, they still can be rich sources of natural bioactive compounds, especially in the fruit and vegetable industry. Recently, much attention has been paid to the use of emerging technologies, including PEF, for the recovery of these compounds. For instance, Ghosh et al. (2019) proposed combining PEF with mechanical pressing for extraction of functional molecules from the waste chicken breast muscle. Andreou et al. (2020) applied PEF in tomato processing to enhance valorization of tomato waste. They noticed the increase in extraction yield of carotenoid up to 56.4%. Lycopene extraction also increased (from 9.84 mg lycopene/100 g to 14.31 mg/100 g tomato residue) for a PEF treatment at 1.0 kV/cm for 7.5 ms. The concentration of extracted total phenolic compounds doubled when tomato waste was treated with a 2 kV/cm and 700 pulses. Other exemplary studies concern the application of PEF for enhancing the extraction of polyphenols from lemon peel residues (Peiró et al. 2019), potato peels (Frontuto et al. 2019), mango and papaya by-products (Parniakov et al. 2016b).

## Impact of PEF on nutrients and bioactive compounds

Studies published recently have been performed mainly in plant-based products, especially juices. They have shown that PEF treatment can be regarded as safe for such bioactive compounds as vitamins, carothenoids and polyphenols. No significant changes in content of vitamin C were reported for apple juice (200, 300, and 400 pulses, electric field strength 30 kV/cm) (Dziadek et al. 2019), pineapple juice (20, 30 and 40 kV and frequency 10, 20, 30 and 40 kHz) (Indriani et al. 2019) or blueburry juice (350 V) (Zhu et al. 2019). It seems that PEF has no impact on the bioaccessibility of this vitamin (Rodríguez-Roque et al. 2015). Salvia-Trujillo et al. (2011), who studied the effect of PEF processing on content of B vitamins in a beverage containing fruit juices (orange, kiwi, mango, and pineapple) and whole and skim milk, found that niacin and thiamin contents in the fruit beverages were not affected by PEF treatment (electric field strength of 35 kV/cm for 1800 μs, a pulse frequency of 200 Hz, and 4 μs bipolar pulses). The use of PEF for pasteurization has the advantage over heat treatment to preserve bioactive compounds. For example, higher concentrations of phenolic acids and flavonoids were observed in PEF-treated tomato juice and orange juice as compared to the conventional thermally treated samples (Odriozola-Serrano et al. 2008; Agcam et al. 2014). PEF pasteurisation of milk maintains its nutritional value. Studies showed that there was no significant effect of pasteurization in a continuous PEF bench scale system (35 kV/cm field strength with 64 pulses of bipolar square wave for 188 μs) on proteins and total solids in milk (Michalac et al. 2003). Similarly, no changes were detected in the retention of thiamine, riboflavin, retinol, cholecalciferol and α-tocopherol in skim milk and fresh bovine whole milk subjected to PEF treatment at 18.3–27.1 kV/cm, 400 ms, at 50–90 °C (Bendicho et al. 2002) or 15–35 kV/cm, 12.5–75 ms at 30 °C (Riener et al., 2009). However, whole milk treated with PEF (20–35 kV/cm, 24–60 ms, 20–40 °C) demonstrated a small reduction of the fat content (Bermúdez-Aguirre et al. 2011). More studies are needed to find out how PEF affects milk protein, as the results published so far are inconsistent. No negative effects on the quality and functionality of oils have been shown in the studies concerning the use of PEF (1.8 kV/cm, 1.6 kJ/kg) for enhancing oil extraction form olives (Andreou et al. 2017). In the case of meat and fish products the number of studies is still too small to draw far-reaching conclusions on the impact of PEF on their nutritional value.

## The potential use of PEF in production of food with increased nutritional value

Pulsed electric fields has been be applied for enrichment of microorganisms in ions essential for proper functioning of human organism (Table 2). Cell biomass prepared in this way may potentially be used for production of functional food. Yeasts are known for their ability to accumulate metal ions from aqueous solutions, e.g. by adsorption and absorption or metabolism (Cha and Cho 2009). Bioaccumulation of metal ions by *Saccharomyces cerevisiae* strains takes place in two stages. In the first stage, biosorption or “passive capture” takes place. It is independent of yeast metabolism and associated with the accumulation of cations on the outer surface of the cell wall. The metal ions are then adsorbed to the anionic sites. The second stage called bioaccumulation or “active capture” is already dependent on the cell metabolism and involves the penetration of metal ions into the cell by means of specific membrane transporters. Metal ions accumulate in vacuoles (MacDiarmid et al. 2002). This mechanism of cation binding may lead to the formation of organic linkages called “bioplexes”. It has been shown that protein and mineral complexes (metaloproteins or bioplexes) are very well assimilated by human organism (De Nicola et al. 2007). The use of yeast or bacteria as a carrier of bioplexes can help to enrich diets with deficient elements such as magnesium, zinc, calcium or selenium. The studies of Pankiewicz and Jamroz (2013), Pankiewicz et al. (2014) showed that yeast cells treated with PEF can accumulate magnesium, zinc and calcium more efficiently due to the phenomenon of electroporation. The authors reported that bioaccumulation of magnesium was 1.5 times higher, zinc—two times higher and calcium even 6 times higher when compared to the culture not treated with PEF. Observations of the PEF-treated yeast cells using laser confocal microscopy revealed that zinc ions are dispersed mainly in cell organelles, and magnesium ions—in the cell wall (Pankiewicz et al. 2015).

**Table 2:** An overview of studies on the application of PEF for increasing biosorption of the selected elements by microorganisms

Microorganism	Nutrient	Conditions of PEF treatment	Effects	References
*Saccharomyces cerevisiae*	Magnesium ions	15-min exposure of the 20-h grown culture to PEF of the 2000 V and pulse width 20 μs; magnesium concentration of 100 μg/mL	Accumulation of magnesium in the yeast biomass reached maximum 3.98 mg/g dm	Pankiewicz and.Jamroz (2010)
*Saccharomyces cerevisiae*	Zinc ions	15 min exposure of the 20 h grown culture to PEFs of 1500 V and 10 μs pulse width; 100 μg Zn/mL medium	Accumulation of zinc in the yeast biomass reached a maximum of 15.57 mg/g d.m (63% higher than in the control)	Pankiewicz and Jamroz (2011)
*Saccharomyces cerevisiae*	Calcium ions	20 min exposure of the 20 h grown culture to PEF of the 5.0 kV/cm and 20 μs pulse width; calcium concentration 100 μg/mL medium	Bioaccumulation of calcium in the yeast biomass reached maximum 2.98 mg/g d.m. It constituted 30% of the total calcium in the medium	Pankiewicz and Jamroz (2013)
*Saccharomyces cerevisiae*	Magnesium and zinc ions	15 min exposure time, culture grown for 20 h field strength of 5.0 kV/cm, pulse width of 20 μs; concentration of 100 µg Mg^2+^/mL and 150 µg Zn^2+^/mL medium	Bioccumulation of magnesium and zinc reached maximum levels of 2.85 and 11.41 mg/g d.m., respectively. Optimization of ion pair concentration and PEF parameters caused a 1.5 or twofold increase of Mg and Zn accumulation, respectively	Pankiewicz et al. (2014)
*Saccharomyces cerevisiae*	Selenium and zinc ions (simultaneously)	Electric field strength of 3 kV/cm and pulse width of 10 μs, treatment of 20-h culture for 10 min; ion con. − 100 μg Se/mL and 150 μg Zn/mL medium	Increase of ions accumulation by 65% for selenium (43.07 mg/g d.m.) and 100% for zinc (14.48 mg/g d.m.)	Pankiewicz et al. (2017)
*Lactobacillus rhamnosus* B 442	Magnesium ions	5 min exposure of the 20 h grown culture to PEF of the 2.0 kV/cm and 20 µs pulse width at conc. 400 μg Mg^2+^/mL medium	PEF caused an increase of magnesium concentration in the cells by 220% in comparison to the control not treated with PEF, accumulation of magnesium in the biomass reached maximum 4.28 mg/g d.m.	Góral, Pankiewicz (2017)
*Lactobacillus* rhamnosus B 442, *Lactobacillus rhamnosus* 1937, and *Lactococcus* lactis JBB 500	Magnesium ions	5 min at pulse width 20 μs, electric field strength 2.0 kV/cm, at the field frequency of 1 Hz; ion conc. of 400 μg Mg^2+^/mL medium	The highest concentration—4.28 mg Mg^2+^/g d.m., was obtained for *L. rhamnosus B* 442. The strains *L. rhamnosus* 1937 and *L. lactis* JBB 500 accumulated, respectively, 1.97 mg Mg^2+^/g d.m. and 1.86 mg Mg^2+^/g d.m.	Góral et al. (2018)
*Lactobacillus rhamnosus* B 442	Zinc ions	Field strength of 3.0 kV/cm, pulse width of 20 µs, electroporation time of 15 min after 20 h of culturing and at zinc conc. of 500 µg/mL medium	Bioaccumulation of zinc increased by 164% compared to the control (no PEF). The maximum content of zinc ions from cells was 2.85 mg Zn^2+^/g d.m. PEF did not reduce bacterial viability or biomass	Góral et al. (2019a, b)

Application of PEF can also enhance bioaccumulation of magnesium ions in *Lactobacillus rhamnosus* B 442, *Lactobacillus rhamnosus* 1937 and *Lactococcus lactis* JBB 500 cells, which can then be used for production of ice cream. The addition of bacteria enriched with Mg^2+^ did not affect the physicochemical characteristics (freezing, fusibility, hardness) of the ice cream and did not change the color of the samples. The higher total number of microorganisms was noted in the ice cream than in the starter cultures, however, the viability of these bacteria was lower than in the control samples (Góral et al. 2018). Góral et al. (2019a, b) used PEF for enhancing bioaccumulation of calcium and zinc in the cells of *Lactobacillus rhamnosus* B 442. The highest bioaccumulation of zinc was observed when the following PEF parameters were applied: field strength 3 kV/cm, pulse width 20 μs and electroporation time of 20 min. The optimal PEF parameters for calcium accumulation were as follows: field strength 3.0 kV/cm, exposure time 10 min, and pulse width 75 µs. Bioaccumulation of Zn^2+^ and Ca^2+^ was higher than in the control sample (with the addition of zinc and without PEF treatment) by, respectively, 164% and 300%. *Lactobacillus rhamnosus* B 442 cells enriched with zinc ions were used for the production of two types of ice cream: unfermented and fermented (Pankiewicz et al. 2019). Also in the case of Se^2+^, the application of pulsed electric fields improves accumulation of this element in yeast cells up to 68% (Pankiewicz et al. 2017).

Results of some studies indicate that the use of PEF during juice production may result in higher content of vitamins and polyphenolic compounds compared to those obtained by traditional technology. Odriozola-Serrano et al. (2008) used PEF (electric field strength of 35 kV/cm, field frequency in the range of 50–250 Hz and pulse width from 1 to 7 μs) to obtain strawberry juice with a higher nutritional value in terms of vitamin C, anthocyanins and antioxidants contents. They observed 98% retention of vitamin C, from 83 to 102% retention of anthocyanins, whereas retention of the antioxidants ranged from 75 to 100%. Maximum retention was obtained when bipolar impulses were applied at field strength of 35 kV/cm, pulse width of 1 μs and frequency of 250 Hz. Cortés et al. (2006) noticed that content of vitamin A in orange juice treated with PEF was higher by 8.1% than in the pasteurized juice. Salvia-Trujillo et al. (2011) showed that beverages containing milk and fruit juice (kiwi, mangoes, oranges and pineapples) treated with high intensity PEF had higher vitamin B2 content than those which were treated thermally. Agcam et al. (2014) in comparative studies of orange juice treated with PEF and thermal pasteurization found that flavonoids and phenolic acids in PEF-treated one were more stable than in juice treated with the thermal pasteurization. PEF also allows the preservation of the initial content of fatty acids and amino acids in the product. Zulueta et al. (2007) did not notice a decrease in the content of saturated, monounsaturated or polyunsaturated fatty acids in orange juice-milk beverage fortified with n − 3 fatty acids and oleic acid processed by high-pulsed electric field. Morales-De La Peña et al. (2012) noted that the content of free amino acids in a fruit juice-soy milk beverage treated with high intensity pulsed electric field and stored at 4 °C was higher than in the beverages thermally treated. On the other hand, the content of histidine, tyrosine, methionine and leucine was lower in the beverages subjected to thermal pasteurization.

PEF can also be used to support the formation of an iron-glycine complex which is stable and has good bioavailability. Zhang et al. (2017) obtained the highest yield of the Fe-glycine complex (81.2%) and the highest iron chelation capacity (107.13 mg/L), using PEF with an electric field strength of 4 kV/cm, frequency of 1 kHz and pulse width of 40 µs for 15 min. The yield value obtained was higher than in the case of the complex formed by thermal treatment (30 min, 60 °C). Based on the results obtained, the authors concluded that PEF can be used in industry to form metal ion complexes with protein amino acids.

Pharmacological effects (e.g. anti-inflammatory, anti-cancer, antioxidant) of C-phycocyanin (C-PC) derived from *Spirulina platensis*, caused this compound to have potential applications in the production of functional foods (Liu et al. 2016). Phycocyanin is a pigment-protein complex with a blue color, which is widely used as a natural food color in the food industry (Taufiqurrahmi et al. 2017). Martinez et al. (2017) used PEF to enhance extraction of C-phycocyanin from *A. platensis*. The purity of the extract obtained from PEF-treated cells was much higher than in the case of other techniques that consisted of complete destruction of the cell.

The development of technology allows the introduction of “novel food” to trade. An example of such food are proteins obtained from microalgae *Ulva* sp. Protein extraction from these microorganisms is possible due to the use of chemical substances, however this method has serious consequences, therefore PEF combined with osmotic shock and mechanical press was used as an alternative extraction method. Subsequently, the extracted proteins were identified and a specific allergen was assigned to them. Extracts that were obtained with PEF contained only one food allergen—superoxide dismutase (SOD), however, more research is needed on the allergenicity of proteins extracted from macroalgae to assess the risk for human consumption (Polokovsky et al. 2019).

## Conclusion

The present review discussed the selected current and potential applications of PEF in food industry. Development of new technologies in food processing is forced, among others, by the growing interest of consumers in fresh-like products of high nutritional value, and the demand for food produced with the use of environmentally friendly methods. PEF is a method that uses electric waves with high voltage amplitude. Short electrical impulses (from microseconds to milliseconds each) of high voltage (typically 10–80 kV/cm) are supplied to the product placed between the electrodes in the chamber. This technology can be used alone or in combination with other methods to obtain products in more energy efficient (e.g. by lowering temperature and time of extraction) and environmentally friendly way. PEF can be applied for pasteurization, enhancement of such processes as drying, freezing, or extraction, but can also support development of functional food containing e.g. easily absorbed ions of elements essential for proper functioning of the human body.

Research of pulsed electric fields technology is carried out around the world. Although this technology has been investigated extensively and there already are commercial PEF systems working in different countries, the majority of the obtained results still refer to the experiments carried out at laboratory scale.
